# Synthesis, crystal structure and Hirshfeld surface analysis of (*E*)-benzo[*d*][1,3]dioxole-5-carbaldehyde oxime

**DOI:** 10.1107/S2056989023004139

**Published:** 2023-05-16

**Authors:** Rengaraj Radhakrishnan, Nour El Hoda Mustaphi, Nada Kheira Sebbar, Joel T. Mague, Aravazhi Amalan Thiruvalluvar

**Affiliations:** a P. G. & Research Department of Physics, Jamal Mohamed College (Autonomous), (affiliated to Bharathidasan University), Tiruchirappalli 620 020, Tamilnadu, India; b Laboratoire Chimie Organique Catalyse et Environnement, Faculté des Sciences, Kenitra, Morocco; c Laboratory of Chemistry and Environment, Applied Bioorganic Chemistry Team, Faculty of Sciences, Ibn Zohr University, Agadir, Morocco; d Department of Chemistry, Tulane University, New Orleans, LA 70118, USA; e Principal (Retired), Kunthavai Naacchiyaar Government Arts College for Women (Autonomous), Thanjavur 613 007, Tamilnadu, India; University of Aberdeen, United Kingdom

**Keywords:** synthesis, crystal structure, benzodioxolane, oxime, O—H⋯N, C—H⋯O, hydrogen bonds, π-stacking, Hirshfeld surface analysis.

## Abstract

The asymmetric unit of the title mol­ecule, C_8_H_7_NO_3_, consists of two mol­ecules differing slightly in conformation and in their inter­molecular inter­actions in the solid. The dihedral angle between the benzene and dioxolane rings is 0.20 (7)° in one mol­ecule and 0.31 (7)° in the other. In the crystal, the two mol­ecules are linked into dimers through pairwise O—H⋯N hydrogen bonds, with these units being formed into stacks by two different sets of aromatic π-stacking inter­actions. The stacks are connected by C—H⋯O hydrogen bonds. A Hirshfeld surface analysis indicates that the most significant contacts in the crystal packing are H⋯O/O⋯H (36.7%), H⋯H (32.2%) and C⋯H/H⋯C (12.7%).

## Abstract 2

The asymmetric unit of the title compound consists of two independent mol­ecules differing slightly in conformation and in their inter­molecular inter­actions in the solid.

## 1. Chemical context

Oxime compounds containing an *R*_2_C=N—OH functional group have been studied for many years because of their important role as acetyl­cholinesterase reactivators and their utility as therapeutic agents for various diseases (Musilek *et al.*, 2011 ▸; Canario *et al.*, 2018 ▸). Various oximes have been identified in plants as biosynthetic inter­mediates and can facilitate a range of processes associated with plant growth and development (Sørensen *et al.*, 2018 ▸). Oximes also have a wide range of biological activities, such as human immunodeficiency virus (HIV) agents that can inhibit HIV protease (Komai *et al.*, 1997 ▸) and can act as anti-inflammatories (Li *et al.*, 2018 ▸; Kwon *et al.*, 2014 ▸). The introduction of an oxime group into an appropriate chemical backbone is a reasonable approach for the preparation of cytotoxic agents and many oxime derivatives have been reported to have therapeutic activity for cancer (Canario *et al.*, 2018 ▸; Shen *et al.*, 2015 ▸) and neurodegenerative disorders (Avrahami *et al.*, 2013 ▸; Yuskaitis *et al.*, 2009 ▸). 
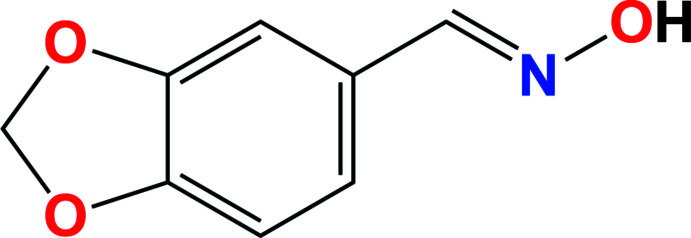


As part of our studies in this area, we now describe the synthesis, structure and Hirshfeld surface analysis of the title compound (I).

## 2. Structural commentary

**Figure 1:**
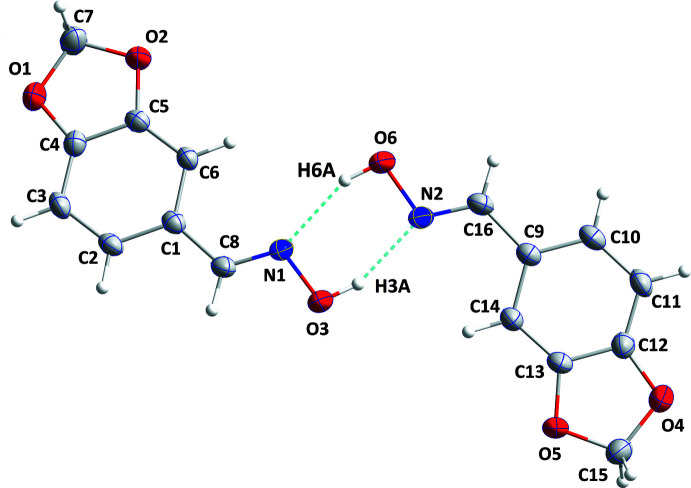
The asymmetric unit with 50% probability ellipsoids. The O—H⋯N hydrogen bonds are depicted by dashed lines.

**Figure 2:**
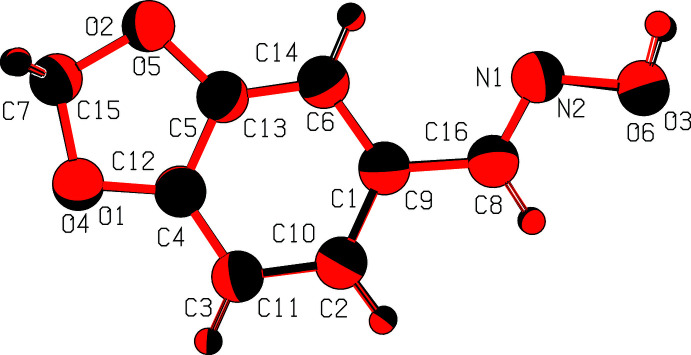
A least-squares overlay of the two independent mol­ecules [inverted O4 mol­ecule (red) on O1 mol­ecule (black)].

The asymmetric unit (Fig. 1 ▸) consists of two independent mol­ecules differing slightly in the orientation of some hydrogen atoms. The benzodioxolane portion of the mol­ecule containing O1 is planar to within 0.0171 (12) Å (r.m.s. deviation of the fitted atoms = 0.0091 Å) with C7 deviating by 0.0171 (12) Å from one side of the mean plane and O1 by 0.0170 (10) Å from the other, indicating a slight twist in the dioxolane ring. The corresponding portion of the second mol­ecule containing O4 is planar to within 0.0041 (11) Å (r.m.s. deviation of the fitted atoms = 0.0030 Å), indicating a conformational difference, albeit small, between the two mol­ecules. The overlay fit of inverted mol­ecule 2 on mol­ecule 1 is shown in Fig. 2 ▸ with the weighted r.m.s. fit of the 12 non-H atoms being 0.036 Å and showing the major differences to be in the hydrogen-atom positions. The C6—C1—C8—N1 and C1—C8—N1—O3 torsion angles are, respectively, 3.9 (2) and −179.96 (11)°, indicating the side chain to be nearly coplanar with the benzodioxolane unit. The corresponding torsion angles in the second mol­ecule are virtually the same as above. The two mol­ecules are connected into dimers through O3—H3*A*⋯N2 and O6—H6*A*⋯N1 hydrogen bonds (Table 1 ▸ and Fig. 1 ▸), generating 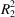 (6) loops.

## 3. Supra­molecular features

**Figure 3:**
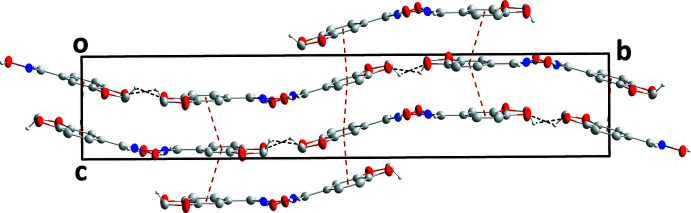
View of the packing seen along the *a*-axis direction with O—H⋯N and C—H⋯O hydrogen bonds and π-stacking inter­actions depicted, respectively, by light blue, black and orange dashed lines.

**Figure 4:**
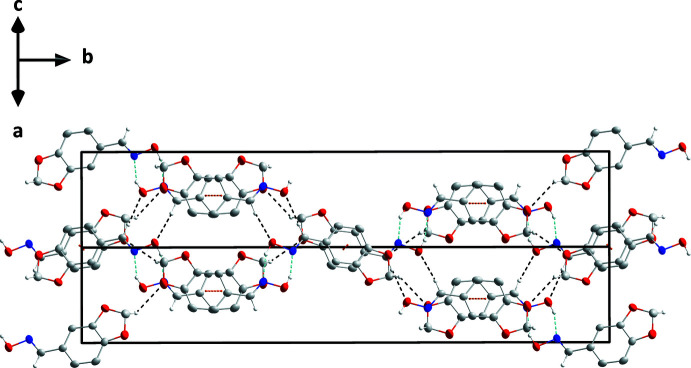
View of the packing seen along the [101] direction. Inter­molecular inter­actions are depicted as in Fig. 3 ▸.

In the crystal, the dimers are connected into stacks extending along the [101] direction through slipped π-stacking inter­actions between the six-membered (*Cg*2: C1–C6 and *Cg*5: C9–C14) rings. For the C1–C6 rings, the centroid–centroid distance is 3.6024 (11) Å with a slippage of 1.185 Å between mol­ecules at *x*, *y*, *z* and −*x*, −*y* + 1, −*z*. These paired mol­ecules make weak, slipped π-stacking inter­actions with corresponding pairs at −*x* + 1, −*y* + 1, −*z* + 1 with a centroid–centroid distance of 3.8479 (11) Å and a slippage of 1.947 Å. The C9–C14 ring has slipped π-stacking inter­actions with its counterparts in mol­ecules at *x* − 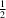 , −*y* + 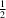 , *z* − 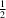 and at *x* + 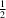 , −*y* + 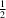 , *z* + 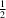 with centroid–centroid distances of 3.8380 (11) Å and dihedral angles of 2.41 (6)° for both. The slippages for these inter­actions (Fig. 3 ▸) are 1.572 and 1.662 Å, respectively. These differences in the π-stacking inter­actions also support the independence of the two mol­ecules in the asymmetric unit. The stacks are associated through C7—H7*B*⋯O4, C8—H8⋯O6, C15—H15*A*⋯O1 and C16—H16⋯O3 hydrogen bonds (Table 1 ▸ and Fig. 4 ▸).

## 4. Database survey

A search using CCDC ConQuest of the Cambridge Structural Database (CSD, Version 5.44, updated to April 2023; Groom *et al.*, 2016 ▸) using the title mol­ecule with all hydrogen atoms deleted gave 26 hits. Most of these contain the search fragment as part of a larger, often polycyclic mol­ecule, but three are considered similar to (I). These are *N*-[1-(2,2-dimethyl-2*H*-1,3-benzodioxol-5-yl)-2-(1*H*-imidazol-1-yl)ethyl­idene]hydroxyl­amine (CSD refcode: GAVWUZ; Ren *et al.*, 2022 ▸), in which the benzo[*d*][1,3]dioxole unit is similar to that in (I), 1-(1,3-benzodioxol-5-yl)-*N*-hy­droxy-3-(1*H*-imidazol-1-yl)propan-1-imine iso­propanol solvate (QEKMAX; Al-Wabli *et al.*, 2017 ▸), in which the benzo[*d*][1,3]dioxole-5-carbaldehyde­oxime takes a (Z) form and (*Z*)-3,4-methyl­ene­dioxy­benzaldehyde oximium 4-toluene­sulfonate (VADDIN; Jerslev *et al.*, 1988 ▸), in which the benzo[*d*][1,3]dioxole unit is similar to that in (I).

## 5. Hirshfeld surface analysis

**Figure 5:**
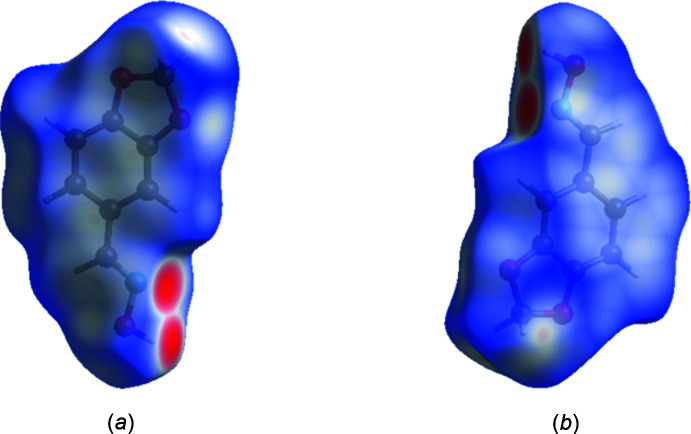
The Hirshfeld surface plots for (I): (*a*) *d*_norm_ for the O1-containing mol­ecule; (*b*) *d*_norm_ for the O4-containing mol­ecule.

**Figure 6:**
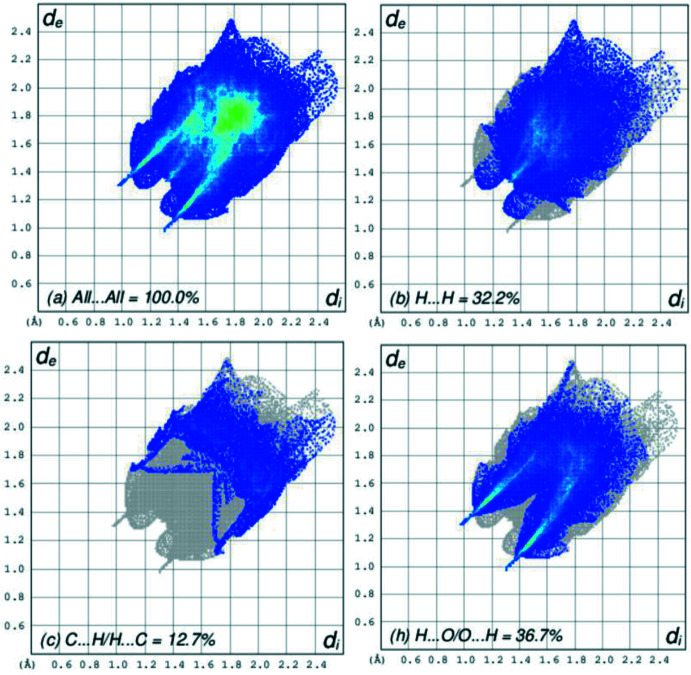
Fingerprint plots for (I) (both mol­ecules): (*a*) all inter­actions; (*b*) H⋯H; (*c*) C⋯H/H⋯C and (*h*) H⋯O/O⋯H.

The Hirshfeld surface analysis was performed with *Crystal Explorer* (Version 21.5; Spackman *et al.*, 2021 ▸). Fig. 5 ▸ shows views of the *d*_norm_ surfaces for the two mol­ecules in the asymmetric unit plotted over the limits from −0.63 to 1.18 a.u for mol­ecule 1 and −0.63 to 1.07 a.u for mol­ecule 2. The O—H⋯N hydrogen bonds, which generate the dimers are indicated by the bright-red spots in Fig. 5 ▸(*a*) and 5(*b*), respectively. Fig. 6 ▸ presents the two-dimensional fingerprint plots involving all inter­molecular inter­actions [Fig. 6 ▸(*a*)] and delineated into C⋯H/H⋯C [Fig. 6 ▸(*c*)], and H⋯O/O⋯H [Fig. 6 ▸(*h*)] inter­actions. For completeness, the H⋯H inter­actions constitute 32.2% of the surface [Fig. 6 ▸(*b*)]. The other inter­actions contribute small amounts, *viz*., C⋯N/N⋯C (1.0%), C⋯O/O⋯C (2.4%), C⋯C (9.5%), H⋯N/N⋯H (4.1%), N⋯O/O⋯N (1.1%), N⋯N (0.0%) and O⋯O (0.4%).

## 6. Synthesis and crystallization

A solution of 5.0 g of sodium hydroxide dissolved in 20 ml of water was mixed with 8.0 g of hydroxyl­amine hydro­chloride dissolved in 15 ml of water, then 8.0 g of benzo[*d*][1,3]dioxole-5-carbaldehyde dissolved in 50 ml of ethanol was added to the mixture. After 5 h of stirring at 273 K, the product was allowed to precipitate and then filtered with a yield of 90%. Single crystals were recrystallized from ethanol solution.

## 7. Refinement

Crystal data, data collection and structure refinement details are summarized in Table 2 ▸. H atoms attached to carbon were placed in calculated positions (C—H = 0.95–0.99 Å) while those attached to oxygen were placed in locations derived from a difference map and their coordinates adjusted to give O—H = 0.87 Å. All were included as riding contributions with isotropic displacement parameters 1.2–1.5 times those of the attached atoms.

## Supplementary Material

Crystal structure: contains datablock(s) I. DOI: 10.1107/S2056989023004139/hb8064sup1.cif

Structure factors: contains datablock(s) I. DOI: 10.1107/S2056989023004139/hb8064Isup2.hkl

Supporting information file. DOI: 10.1107/S2056989023004139/hb8064Isup3.cdx

Supporting information file. DOI: 10.1107/S2056989023004139/hb8064Isup4.cml

CCDC reference: 2262070

Additional supporting information:  crystallographic information; 3D view; checkCIF report

## Figures and Tables

**Table 1:** Hydrogen-bond geometry (Å, °)

*D*—H⋯*A*	*D*—H	H⋯*A*	*D*⋯*A*	*D*—H⋯*A*
O3—H3*A*⋯N2	0.87	1.93	2.7549 (16)	158
C7—H7*B*⋯O4^i^	0.99	2.58	3.239 (2)	124
C8—H8⋯O6^ii^	0.95	2.43	3.3754 (18)	173
O6—H6*A*⋯N1	0.87	1.97	2.7989 (17)	158
C15—H15*A*⋯O1^iii^	0.99	2.54	3.1775 (19)	122
C16—H16⋯O3^iv^	0.95	2.59	3.5173 (18)	167

Symmetry codes: (i) 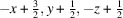 ; (ii) 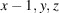 ; (iii) 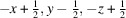 ; (iv) 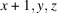 .

**Table 2:** Experimental details

Crystal data	
Chemical formula	C_8_H_7_NO_3_
*M*_r_	165.15
Crystal system, space group	Monoclinic, *P*2_1_/*n*
Temperature (K)	150
*a*, *b*, *c* (Å)	6.8724 (14), 33.502 (7), 7.3449 (15)
β (°)	117.238 (3)
*V* (Å^3^)	1503.6 (5)
*Z*	8
Radiation type	Mo *K*α
μ (mm^−1^)	0.11
Crystal size (mm)	0.36 × 0.17 × 0.10

Data collection	
Diffractometer	Bruker SMART APEX CCD
Absorption correction	Multi-scan (*SADABS*; Krause *et al.*, 2015 ▸)
*T*_min_, *T*_max_	0.82, 0.99
No. of measured, independent and observed [*I* > 2σ(*I*)] reflections	28032, 3858, 2836
*R*_int_	0.048
(sin θ/λ)_max_ (Å^−1^)	0.675

Refinement	
*R*[*F* ^2^ > 2σ(*F* ^2^)], *wR*(*F* ^2^), *S*	0.046, 0.127, 1.05
No. of reflections	3858
No. of parameters	217
H-atom treatment	H-atom parameters constrained
Δρ_max_, Δρ_min_ (e Å^−3^)	0.31, −0.19

Computer programs: *APEX3* and *SAINT* (Bruker, 2016 ▸), *SHELXT* (Sheldrick, 2015*a* ▸), *SHELXL2018/3* (Sheldrick, 2015*b* ▸), *DIAMOND* (Brandenburg & Putz, 2012 ▸), *PLATON* (Spek, 2020 ▸) and *publCIF* (Westrip, 2010 ▸).

## supplementary crystallographic information

### Crystal data

**Table:**

C_8_H_7_NO_3_	*F*(000) = 688
*M**_r_* = 165.15	*D*_x_ = 1.459 Mg m^−^^3^
Monoclinic, *P*2_1_/*n*	Mo *K*α radiation, λ = 0.71073 Å
*a* = 6.8724 (14) Å	Cell parameters from 6661 reflections
*b* = 33.502 (7) Å	θ = 2.4–28.1°
*c* = 7.3449 (15) Å	µ = 0.11 mm^−^^1^
β = 117.238 (3)°	*T* = 150 K
*V* = 1503.6 (5) Å^3^	Plate, colourless
*Z* = 8	0.36 × 0.17 × 0.10 mm

### Data collection

**Table:**

Bruker SMART APEX CCD diffractometer	3858 independent reflections
Radiation source: fine-focus sealed tube	2836 reflections with *I* > 2σ(*I*)
Graphite monochromator	*R*_int_ = 0.048
Detector resolution: 8.3333 pixels mm^-1^	θ_max_ = 28.7°, θ_min_ = 2.4°
φ and ω scans	*h* = −9→9
Absorption correction: multi-scan (*SADABS*; Krause *et al.*, 2015)	*k* = −45→43
*T*_min_ = 0.82, *T*_max_ = 0.99	*l* = −9→9
28032 measured reflections	

### Refinement

**Table:**

Refinement on *F*^2^	Primary atom site location: dual
Least-squares matrix: full	Secondary atom site location: difference Fourier map
*R*[*F*^2^ > 2σ(*F*^2^)] = 0.046	Hydrogen site location: mixed
*wR*(*F*^2^) = 0.127	H-atom parameters constrained
*S* = 1.05	*w* = 1/[σ^2^(*F*_o_^2^) + (0.0621*P*)^2^ + 0.2227*P*] where *P* = (*F*_o_^2^ + 2*F*_c_^2^)/3
3858 reflections	(Δ/σ)_max_ < 0.001
217 parameters	Δρ_max_ = 0.31 e Å^−^^3^
0 restraints	Δρ_min_ = −0.19 e Å^−^^3^

### Special details

**Table:**

Experimental. The diffraction data were obtained from 3 sets of 400 frames, each of width 0.5° in ω, collected at φ = 0.00, 90.00 and 180.00° and 2 sets of 800 frames, each of width 0.45° in φ, collected at ω = –30.00 and 210.00°. The scan time was 20 sec/frame.
Geometry. All esds (except the esd in the dihedral angle between two l.s. planes) are estimated using the full covariance matrix. The cell esds are taken into account individually in the estimation of esds in distances, angles and torsion angles; correlations between esds in cell parameters are only used when they are defined by crystal symmetry. An approximate (isotropic) treatment of cell esds is used for estimating esds involving l.s. planes.
Refinement. Refinement of F^2^ against ALL reflections. The weighted R-factor wR and goodness of fit S are based on F^2^, conventional R-factors R are based on F, with F set to zero for negative F^2^. The threshold expression of F^2^ > 2sigma(F^2^) is used only for calculating R-factors(gt) etc. and is not relevant to the choice of reflections for refinement. R-factors based on F^2^ are statistically about twice as large as those based on F, and R- factors based on ALL data will be even larger. H-atoms attached to carbon were placed in calculated positions (C—H = 0.95 - 0.99 Å) while those attached to oxygen were placed in locations derived from a difference map and their coordinates adjusted to give O—H = 0.87 Å. All were included as riding contributions with isotropic displacement parameters 1.2 - 1.5 times those of the attached atoms.

### Fractional atomic coordinates and isotropic or equivalent isotropic displacement parameters (Å^2^)

**Table:**

	*x*	*y*	*z*	*U*_iso_*/*U*_eq_	
O1	0.21075 (18)	0.58110 (3)	0.12890 (17)	0.0385 (3)	
O2	0.50131 (18)	0.54433 (3)	0.13879 (17)	0.0377 (3)	
O3	0.40957 (17)	0.36128 (3)	0.45505 (17)	0.0377 (3)	
H3A	0.516808	0.349351	0.445154	0.057*	
N1	0.43129 (18)	0.40031 (3)	0.39281 (18)	0.0287 (3)	
C1	0.2551 (2)	0.46413 (4)	0.30915 (19)	0.0254 (3)	
C2	0.0814 (2)	0.48668 (4)	0.3030 (2)	0.0290 (3)	
H2	−0.020388	0.474321	0.339202	0.035*	
C3	0.0524 (2)	0.52685 (4)	0.2452 (2)	0.0316 (3)	
H3	−0.065828	0.542093	0.241589	0.038*	
C4	0.2043 (2)	0.54306 (4)	0.1939 (2)	0.0280 (3)	
C5	0.3775 (2)	0.52084 (4)	0.1994 (2)	0.0262 (3)	
C6	0.4081 (2)	0.48172 (4)	0.25517 (19)	0.0252 (3)	
H6	0.527136	0.466906	0.257669	0.030*	
C7	0.4031 (3)	0.58311 (4)	0.0990 (3)	0.0379 (4)	
H7A	0.363159	0.591287	−0.043339	0.045*	
H7B	0.507199	0.602939	0.193416	0.045*	
C8	0.2711 (2)	0.42252 (4)	0.3722 (2)	0.0285 (3)	
H8	0.159251	0.411565	0.398925	0.034*	
O4	0.79029 (19)	0.15551 (3)	0.4126 (2)	0.0489 (3)	
O5	0.54405 (19)	0.19665 (3)	0.4559 (2)	0.0488 (3)	
O6	0.84324 (18)	0.38394 (3)	0.42029 (17)	0.0392 (3)	
H6A	0.725059	0.395443	0.409864	0.059*	
N2	0.78297 (19)	0.34360 (4)	0.42083 (18)	0.0299 (3)	
C9	0.8894 (2)	0.27623 (4)	0.4103 (2)	0.0266 (3)	
C10	1.0360 (2)	0.25112 (4)	0.3838 (2)	0.0319 (3)	
H10	1.152810	0.262583	0.366388	0.038*	
C11	1.0166 (2)	0.20967 (5)	0.3820 (2)	0.0354 (3)	
H11	1.116764	0.192674	0.363450	0.043*	
C12	0.8452 (2)	0.19468 (4)	0.4082 (2)	0.0320 (3)	
C13	0.6986 (2)	0.21941 (4)	0.4347 (2)	0.0302 (3)	
C14	0.7148 (2)	0.25990 (4)	0.4373 (2)	0.0292 (3)	
H14	0.613289	0.276437	0.456418	0.035*	
C15	0.5989 (3)	0.15609 (4)	0.4420 (2)	0.0369 (3)	
H15A	0.476274	0.142790	0.325521	0.044*	
H15B	0.626735	0.141616	0.569104	0.044*	
C16	0.9197 (2)	0.31931 (4)	0.4083 (2)	0.0299 (3)	
H16	1.043047	0.329466	0.397484	0.036*	

### Atomic displacement parameters (Å^2^)

**Table:**

	*U*^11^	*U*^22^	*U*^33^	*U*^12^	*U*^13^	*U*^23^
O1	0.0392 (6)	0.0285 (6)	0.0509 (7)	0.0074 (4)	0.0234 (5)	0.0055 (5)
O2	0.0400 (6)	0.0297 (6)	0.0560 (7)	0.0021 (4)	0.0328 (5)	0.0076 (5)
O3	0.0355 (6)	0.0243 (5)	0.0599 (7)	−0.0013 (4)	0.0277 (5)	0.0051 (5)
N1	0.0266 (6)	0.0231 (6)	0.0357 (6)	−0.0034 (4)	0.0137 (5)	−0.0002 (5)
C1	0.0204 (6)	0.0296 (7)	0.0245 (6)	−0.0017 (5)	0.0088 (5)	−0.0042 (5)
C2	0.0205 (7)	0.0358 (8)	0.0324 (7)	−0.0030 (5)	0.0137 (6)	−0.0045 (6)
C3	0.0223 (7)	0.0361 (8)	0.0373 (8)	0.0048 (6)	0.0144 (6)	−0.0047 (6)
C4	0.0270 (7)	0.0264 (7)	0.0282 (7)	0.0034 (5)	0.0105 (6)	−0.0011 (5)
C5	0.0240 (7)	0.0308 (7)	0.0257 (6)	−0.0017 (5)	0.0131 (5)	−0.0023 (5)
C6	0.0212 (6)	0.0281 (7)	0.0275 (7)	0.0020 (5)	0.0121 (5)	−0.0020 (5)
C7	0.0463 (9)	0.0327 (8)	0.0414 (9)	0.0046 (7)	0.0259 (7)	0.0068 (6)
C8	0.0229 (7)	0.0298 (7)	0.0343 (7)	−0.0043 (5)	0.0143 (6)	−0.0035 (6)
O4	0.0423 (7)	0.0275 (6)	0.0825 (9)	0.0000 (5)	0.0335 (7)	−0.0056 (5)
O5	0.0397 (6)	0.0279 (6)	0.0940 (10)	−0.0066 (5)	0.0439 (7)	−0.0038 (6)
O6	0.0347 (6)	0.0276 (6)	0.0612 (7)	−0.0021 (4)	0.0269 (5)	0.0071 (5)
N2	0.0272 (6)	0.0265 (6)	0.0360 (6)	−0.0026 (5)	0.0145 (5)	0.0037 (5)
C9	0.0208 (6)	0.0313 (7)	0.0277 (7)	−0.0005 (5)	0.0110 (5)	0.0000 (5)
C10	0.0214 (7)	0.0408 (9)	0.0354 (8)	0.0005 (6)	0.0147 (6)	−0.0002 (6)
C11	0.0240 (7)	0.0399 (9)	0.0435 (8)	0.0058 (6)	0.0165 (6)	−0.0040 (6)
C12	0.0265 (7)	0.0287 (8)	0.0377 (8)	0.0025 (5)	0.0119 (6)	−0.0026 (6)
C13	0.0211 (7)	0.0319 (8)	0.0380 (8)	−0.0029 (5)	0.0139 (6)	−0.0017 (6)
C14	0.0227 (7)	0.0296 (7)	0.0371 (8)	0.0011 (5)	0.0154 (6)	−0.0017 (6)
C15	0.0358 (8)	0.0286 (8)	0.0441 (9)	−0.0017 (6)	0.0164 (7)	0.0016 (6)
C16	0.0237 (7)	0.0357 (8)	0.0331 (7)	−0.0029 (6)	0.0153 (6)	0.0023 (6)

### Geometric parameters (Å, º)

**Table:**

O1—C4	1.3685 (17)	O4—C12	1.3699 (18)
O1—C7	1.4377 (19)	O4—C15	1.427 (2)
O2—C5	1.3742 (16)	O5—C13	1.3730 (17)
O2—C7	1.4311 (17)	O5—C15	1.4264 (18)
O3—N1	1.4154 (15)	O6—N2	1.4140 (15)
O3—H3A	0.8702	O6—H6A	0.8701
N1—C8	1.2790 (18)	N2—C16	1.2776 (18)
C1—C2	1.3957 (18)	C9—C10	1.3931 (19)
C1—C6	1.4110 (18)	C9—C14	1.4129 (18)
C1—C8	1.4570 (19)	C9—C16	1.4595 (19)
C2—C3	1.398 (2)	C10—C11	1.394 (2)
C2—H2	0.9500	C10—H10	0.9500
C3—C4	1.373 (2)	C11—C12	1.373 (2)
C3—H3	0.9500	C11—H11	0.9500
C4—C5	1.3890 (19)	C12—C13	1.3849 (19)
C5—C6	1.3603 (19)	C13—C14	1.3606 (19)
C6—H6	0.9500	C14—H14	0.9500
C7—H7A	0.9900	C15—H15A	0.9900
C7—H7B	0.9900	C15—H15B	0.9900
C8—H8	0.9500	C16—H16	0.9500
			
C4—O1—C7	106.02 (11)	C12—O4—C15	105.87 (11)
C5—O2—C7	106.39 (11)	C13—O5—C15	106.11 (11)
N1—O3—H3A	100.3	N2—O6—H6A	99.2
C8—N1—O3	111.31 (11)	C16—N2—O6	112.48 (11)
C2—C1—C6	120.04 (13)	C10—C9—C14	120.06 (13)
C2—C1—C8	117.85 (12)	C10—C9—C16	118.69 (12)
C6—C1—C8	122.10 (12)	C14—C9—C16	121.24 (12)
C1—C2—C3	122.15 (13)	C9—C10—C11	122.04 (13)
C1—C2—H2	118.9	C9—C10—H10	119.0
C3—C2—H2	118.9	C11—C10—H10	119.0
C4—C3—C2	116.33 (12)	C12—C11—C10	116.57 (13)
C4—C3—H3	121.8	C12—C11—H11	121.7
C2—C3—H3	121.8	C10—C11—H11	121.7
O1—C4—C3	127.89 (13)	O4—C12—C11	128.09 (13)
O1—C4—C5	110.19 (12)	O4—C12—C13	110.11 (13)
C3—C4—C5	121.91 (13)	C11—C12—C13	121.80 (14)
C6—C5—O2	128.03 (12)	C14—C13—O5	127.91 (12)
C6—C5—C4	122.51 (12)	C14—C13—C12	122.58 (13)
O2—C5—C4	109.45 (12)	O5—C13—C12	109.51 (13)
C5—C6—C1	117.05 (12)	C13—C14—C9	116.94 (12)
C5—C6—H6	121.5	C13—C14—H14	121.5
C1—C6—H6	121.5	C9—C14—H14	121.5
O2—C7—O1	107.86 (11)	O5—C15—O4	108.40 (12)
O2—C7—H7A	110.1	O5—C15—H15A	110.0
O1—C7—H7A	110.1	O4—C15—H15A	110.0
O2—C7—H7B	110.1	O5—C15—H15B	110.0
O1—C7—H7B	110.1	O4—C15—H15B	110.0
H7A—C7—H7B	108.4	H15A—C15—H15B	108.4
N1—C8—C1	122.00 (12)	N2—C16—C9	121.07 (12)
N1—C8—H8	119.0	N2—C16—H16	119.5
C1—C8—H8	119.0	C9—C16—H16	119.5
			
C6—C1—C2—C3	−0.3 (2)	C14—C9—C10—C11	0.2 (2)
C8—C1—C2—C3	179.76 (13)	C16—C9—C10—C11	−179.38 (13)
C1—C2—C3—C4	0.2 (2)	C9—C10—C11—C12	−0.2 (2)
C7—O1—C4—C3	179.08 (14)	C15—O4—C12—C11	−179.48 (15)
C7—O1—C4—C5	−2.05 (15)	C15—O4—C12—C13	0.38 (16)
C2—C3—C4—O1	178.64 (13)	C10—C11—C12—O4	−179.98 (14)
C2—C3—C4—C5	−0.1 (2)	C10—C11—C12—C13	0.2 (2)
C7—O2—C5—C6	−179.40 (14)	C15—O5—C13—C14	179.94 (14)
C7—O2—C5—C4	1.47 (15)	C15—O5—C13—C12	0.07 (17)
O1—C4—C5—C6	−178.80 (12)	O4—C12—C13—C14	179.83 (13)
C3—C4—C5—C6	0.1 (2)	C11—C12—C13—C14	−0.3 (2)
O1—C4—C5—O2	0.38 (16)	O4—C12—C13—O5	−0.29 (17)
C3—C4—C5—O2	179.33 (12)	C11—C12—C13—O5	179.58 (14)
O2—C5—C6—C1	−179.25 (13)	O5—C13—C14—C9	−179.49 (14)
C4—C5—C6—C1	−0.2 (2)	C12—C13—C14—C9	0.4 (2)
C2—C1—C6—C5	0.29 (19)	C10—C9—C14—C13	−0.3 (2)
C8—C1—C6—C5	−179.76 (12)	C16—C9—C14—C13	179.28 (13)
C5—O2—C7—O1	−2.71 (15)	C13—O5—C15—O4	0.16 (17)
C4—O1—C7—O2	2.92 (15)	C12—O4—C15—O5	−0.33 (16)
O3—N1—C8—C1	−179.96 (11)	O6—N2—C16—C9	178.85 (11)
C2—C1—C8—N1	−176.17 (13)	C10—C9—C16—N2	176.16 (13)
C6—C1—C8—N1	3.9 (2)	C14—C9—C16—N2	−3.5 (2)

### Hydrogen-bond geometry (Å, º)

**Table:**

*D*—H···*A*	*D*—H	H···*A*	*D*···*A*	*D*—H···*A*
O3—H3*A*···N2	0.87	1.93	2.7549 (16)	158
C7—H7*B*···O4^i^	0.99	2.58	3.239 (2)	124
C8—H8···O6^ii^	0.95	2.43	3.3754 (18)	173
O6—H6*A*···N1	0.87	1.97	2.7989 (17)	158
C15—H15*A*···O1^iii^	0.99	2.54	3.1775 (19)	122
C16—H16···O3^iv^	0.95	2.59	3.5173 (18)	167

Symmetry codes: (i) −*x*+3/2, *y*+1/2, −*z*+1/2; (ii) *x*−1, *y*, *z*; (iii) −*x*+1/2, *y*−1/2, −*z*+1/2; (iv) *x*+1, *y*, *z*.
